# Impact of MLC leaf width on volumetric‐modulated arc therapy planning for head and neck cancers

**DOI:** 10.1120/jacmp.v14i6.4074

**Published:** 2013-11-08

**Authors:** Caroline Lafond, Enrique Chajon, Anne Devillers, Guillaume Louvel, Sandra Toublanc, Mickael Olivier, Antoine Simon, Renaud de Crevoisier, Jean‐Pierre Manens

**Affiliations:** ^1^ Centre Eugène Marquis Rennes France; ^2^ INSERM U1099 Rennes France; ^3^ Laboratoire du Traitement du Signal et de l'Image University of Rennes Rennes France

**Keywords:** VMAT, dosimetry, head and neck cancer, multileaf collimators, leaf width

## Abstract

This dosimetric study investigated the impact of multileaf collimators (MLC) leaf width in volumetric‐modulated arc therapy (VMAT) for head and neck cancers (HNC), either with a “standard” simultaneously integrated boost technique (S‐SIB) or with a “dose painting” SIB technique (DP‐SIB). HNC patients were planned either with an S‐SIB comprising three dose levels, from 56 to 70 Gy (16 patients), or with a DP‐SIB comprising five dose levels, from 56 to 84 Gy (8 patients), in 35 fractions. Two VMAT plans were calculated for each SIB technique using two Elekta MLCs: MLCi2 with 10 mm leaf width and Beam Modulator (BM) with 4 mm leaf width. Dose distributions were evaluated by comparing doses on PTVs, main OARs, and healthy tissue, and by comparing conformation indexes. Treatment efficiencies were evaluated by comparing the number of monitor units and the number of needed arcs. Comparisons of the two MLCs depending on the two SIB techniques showed: i) Regarding PTVs: Dmean and D2% on lower doses PTV decreased respectively by 0.5 Gy (p=0.01) and 0.9 Gy (p=0.01) with BM than with MLCi2 for S‐SIB; no significant difference was found for DP‐SIB; ii) Regarding OARs: for spinal cord and brainstem, D2% decreased respectively by 1.2 Gy (p=0.03) and 4.2 Gy (p=0.04) with BM than with MLCi2 for S‐SIB; for controlateral parotid, D50% decreased by 1.5 Gy (p=0.01) with BM than with MLCi2 for S‐SIB; iii) Regarding treatment efficiency : the number of monitor units was 44% (p=0.00) and 51% (p=0.01) higher with BM for S‐SIB and DP‐SIB, respectively. Two arcs were more frequently needed with BM to reach an acceptable dose distribution. This study demonstrated that Beam Modulator (4 mm leaf width) and MLCi2 (10 mm leaf width) MLCs from Elekta provided satisfactory dose distributions for treatment delivery with VMAT technique for complex HNC cases with standard and dose painting prescriptions. OAR sparing was better with BM, mainly for brainstem and spinal cord. However, delivery efficiency of VMAT plans was better with MLCi2.

PACS numbers: 87.56.N‐, 87.56.nk, 87.55.D‐

## I. INTRODUCTION

Radiotherapy is one of the crucial components of the modern multidisciplinary approach of head and neck cancer (HNC) treatment. Intensity‐modulated radiotherapy (IMRT) provides the benefit of delivering conformal doses to the target volume while maintaining low doses to the critical organs.^(1)^

Karl Otto has recently introduced the volumetric‐modulated arc therapy (VMAT) technique.^(2)^ VMAT is a natural progression of IMRT delivery, combining the possibility to vary simultaneously the dose rate, the speed of gantry rotation, and the multileaf collimator (MLC) shape. Several studies showed that VMAT technique can achieve at least equivalent, and possibly superior, dose distributions than step‐and‐shoot (S&S) or sliding widow (SW) IMRT. Moreover, efficiency of the treatment is often improved with the VMAT technique by decreasing significantly the number of monitor units (MU) and delivery time.^3^, ^4^, ^5^, ^6^, ^7^, ^8^

A few studies investigated the impact of MLC leaf width on IMRT techniques for HNC. Zwicker et al.^(9)^ have proved the advantage to use narrower MLC leaf width for HNC in S&S IMRT. Wang et al.^(10)^ have investigated the impact of MLC leaf width for nasopharyngeal cancer in S&S technique. They showed that narrower MLC leaf width only improved dose coverage and treatment efficiency. No previous study investigated the impact of MLC leaf width on VMAT technique for HNC using specifically simultaneous integrated boost (SIB) prescriptions. Only one recent study compared two MLC leaf width in VMAT for prostate and rectum cancer with a favorable effect when using more narrow MLC leaf width.^(11)^

For HNC, IMRT could be used to increase local control by escalating the dose in the most radio‐resistant or proliferative part of the tumor.^(12)^ Technical feasibility of 18F‐fluorodesoxyglucose positron emission tomography (FDG‐PET) imaging dose painting has been recently shown by different groups.^13^, ^14^ Dose levels are usually arbitrarily determined. Madani et al.^(15)^ showed in a phase I dose escalation trial the feasibility of delivering two dose levels: 72.5 Gy (78.2 Gy NID2Gy (normalized isoeffective dose)) and 77.5 Gy (86.7 Gy NID2Gy). Following this principle, we explored the influence of multileaf collimator in a theoretical scenario of dose painting requiring multiple target subvolumes defined by FDG‐PET.

The aim of this study was to investigate the impact of MLC leaf width on VMAT dose distribution for head and neck cancer (HNC) using two different SIB prescriptions. The first prescription, considered as “standard”, comprised three dose levels (S‐SIB). The second, considered as a more “dose painting” approach, comprised five dose levels (DP‐SIB).

## MATERIALS AND METHODS

### A. Patients

CT scans of 16 locally advanced nonmetastatic HNC cases treated by S&S IMRT with curative intention were selected for this plan comparison study. The primary site was the oropharynx in four cases, the hypopharynx in eight, the oral cavity in three, and the larynx in one case. The American Joint Committee on Cancer (AJCC) staging system was used according to the primary tumor location. The T stage was T1 in one, T2 in 3, T3 in 5, and T4 in seven cases. The N stage was N0 in 6, N1 in 2, N2 in 5, and N3 in three cases.

All patients were immobilized by using a custom Aquaplast mask holding both neck and shoulders. Computed tomography contrast‐enhanced images indexed every 3 mm were acquired extending from the vertex to the carina. All target volumes and OARs were delineated slice by slice on CT images. The definition of target volumes was made in accordance with the ICRU Report 83.^(16)^ The international guidelines for HNC cancer were used to define clinical target volumes (CTV) according to the site of the primary tumor.^(17)^ In brief, the gross target volume (GTV) was defined by clinical examination and computed tomography, FDG‐PET. The CTVs were defined by the GTV plus areas considered containing potential microscopic disease or by the lymph node levels at risk of subclinical disease. The planning target volume (PTV), aimed to account for setup uncertainties, was defined using an additional margin of 5 mm around the CTV.

For eight patients, PET imaging was acquired in the same position as the planning CT scan 60 min after injection of 4 MBq/Kg of FDG. Two metabolic tumor volumes (MTVs) were delineated on a workstation (Advantage Windows; GE Healthcare, Waukesha, WI) using thresholds of 40% and 70% of the maximum tumor uptake, called 40%‐MTV and 70%‐MTV, respectively. Both MTVs were transferred to the treatment planning system (TPS).

### B. Planning objectives

All treatments were planned using an SIB technique.

For S‐SIB technique, the three PTVs were defined as follows: PTV70 encompassed the gross tumor plus a margin of 5 mm; PTV63 was defined as the high‐risk subclinical disease volume plus a 5 mm margin and excluding the PTV70; PTV56 was defined as the low‐risk subclinical disease plus a 5 mm margin and excluding the PTV70 and the PTV63. Each volume was typically treated once daily, five days a week, in 35 fractions (PTV70: 2 Gy/fr for a total dose of 70 Gy; PTV63: 1.8 Gy/fr for a total dose of 63 Gy; PTV56: 1.6 Gy/fr for a total dose of 56 Gy).

For DP‐SIB technique, the two additional PTVs were defined as follows: PTV 84 Gy encompassed the 70%‐MTV plus a margin of 5 mm; and PTV 78 Gy encompassed the 40%‐MTV plus a margin of 5 mm and avoiding the PTV 84 Gy. In case of DP‐SIB, PTV 70 Gy was modified to avoid the PTV 84 Gy and the PTV 78 Gy. The prescribed doses for those subvolumes were 84 Gy (2.4 Gy/fr) and 78 Gy (2.23 Gy/fr) in 35 fractions.

The target dose objectives were: more than 95% of any PTV should receive more than 95% of the prescribed dose or more than 98% of any PTV should receive more than 90% of the prescribed dose; no more than 20% of any PTV could receive more than 110% of the prescribed dose; and no more than 1% or 1 cm^3^ of the tissue outside the PTV should receive more than 110% of the dose prescribed to the primary dose target (PTV 70 Gy). To accept a plan, the rules were: at least two‐thirds of the PTVs had to respect the objectives V95%≥95%, and at least two‐thirds of the PTVs had to respect the objectives V90%≥8%. For inverse planning and evaluation dose, target volumes were limited to avoid the first 3 mm of the build‐up region.^(18)^

The following OAR dose constraints were used: the brainstem and spinal cord maximal doses were 50 Gy and 45 Gy, respectively. Planning objectives were: mean dose <26Gy or the maximal dose received by 50% of the volume <30Gy for the parotid glands.

### C. Collimators specification

The plans were calculated for two Elekta internal collimators: MLCi2 and Beam Modulator (BM) (Elekta, Stockholm, Sweden).

The MLCi2 has 40 pairs of leaves with a 10 mm width at the isocenter. The minimum opposite leaf gap is 0.5 cm and the maximum field size is 40×40cm. The maximum distance between leaves on the same leaf guide is 32.5 cm (12.5 cm over central axis). MLCi2 was used without leaves interdigitation. Under the leaves, there are auto tracking backup diaphragms, which are movable during the treatment, for automated leakage reduction. Perpendicular jaws are also movable during the treatment. The average MLC transmission is 0.60% measured under the leaves and 0.13% measured under the leaves and parallel jaws. The leaves have a rounded leaf end and a flat side.

The BM has 40 pairs of leaves with a width at the isocenter of 4 mm. The minimum opposite leaf gap is 0.05 cm and the maximum field size is 21 cm by 16 cm. The maximum distance between leaves on the same leaf guide is 21 cm (full field travel) and leaves interdigitation is allowed. The average MLC transmission is 0.60% measured under the leaves. There are no additional jaws. The leaves have a rounded leaf end and a flat side.

### D. VMAT plan

All plans were planned with the Philips TPS, Pinnacle3 (version 9.0) (Philips Healthcare, Andover, MA), using a 6 MV photons beams from an Elekta Synergy linear accelerator. Final dose was computed with a collapsed cone algorithm. To achieve the desired dose distribution, VMAT plans used either one full arc in clockwise direction or two full arcs in clockwise and counterclockwise directions in a same plane, according to the rules that have been explained in Materials and Methods section B. Plans were optimized with the SmartArc algorithm.^(19)^ For each patient and each MLC, inverse constraints were optimized. Each arc was made up of 90 control points (CP) separated by 4°. For BM, the collimator angle was set at 90° to have the larger maximal dimension of the MLC (i.e., 21 cm) in the craniocaudal direction. MLC positions, MUs, and dose rate were optimized for each CP. During the delivery, the MLC moved while the gantry rotated at a variable MU/degree. To deliver a variable MU/degree, the treatment control system (TCS) from the linear accelerator optimized the gantry speed and the dose rate to minimize the beam hold‐offs. The leaves of the MLC and the gantry moved linearly between two CP, and leaves and gantry speeds were optimized to get the faster delivery. All linear and rotational movements during radiation had to respect the minimum variations of 0.3 MU/cm and 0. 1 MU/degree, respectively. For our accelerator, the minimum and maximum dose rate allowed is 25 MU/min and 400 MU/min, respectively. The maximum gantry speed is 6° per sec.

### E. Evaluation tools

Mean dose‐volume histograms (DVHs) were computed for PTVs and main OARs. Some specific values of DVH were analyzed for the 16 patients. Considering PTV, maximum dose (D2%), minimum dose (D98%), mean dose (Dmean), and V95% were analyzed. For spinal cord and brainstem, D2% and Dmean were analyzed. For ipsi‐ and controlateral parotids, Dmean, median dose (Dmed), V15Gy, V30Gy, and V45Gy were analyzed. For healthy tissues, defined as external contours minus PTVs, we have analyzed V5Gy, V10Gy, V25Gy, and V50Gy. For skin, defined as superficial region with a 3 mm thickness, Dmean, V5Gy, V10Gy, V25Gy, and V50Gy were analyzed. The target homogeneity was expressed by the Homogeneity Index (HI) defined as (D5%‐D95%)/Dmean. The degree of conformity was measured with the Conformity Index (CI) defined as the ratio between the reference isodose (95% of the prescribed dose) volume and the volume of the PTV.^(20)^ COnformal INdex (COIN), as defined by Baltas,^(21)^ was also calculated. NTCP values were calculated for parotids with the parameters defined by Dijkema et al.^(22)^ and Houweling et al.:^(23)^ TD50=39.9Gy,n=1,m=0.4.

To evaluate the dose delivery efficiency, the numbers of monitor units (MU) were compared.

Statistical analysis used two‐sided Wilcoxon signed‐rank test, a nonparametric test calculated with the IBM software, PASW (version 18.0.0) (IBM, Armonk, NY). A value of p<0.05 was considered statistically significant.

## III. RESULTS

**Figure 1:**
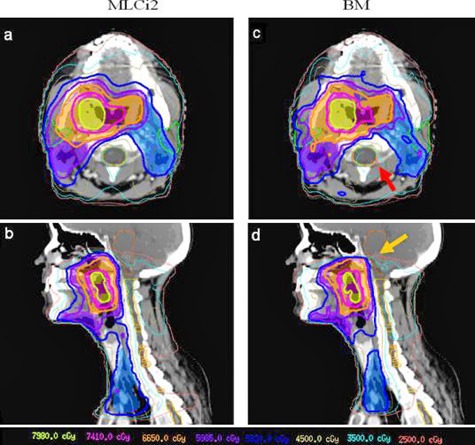
Dose distributions depending on collimator leaf width: 10 mm MLCI2 leaf width ((a), (b)) and 4 mm BM leaf width ((c), (d)) using a “dose painting” simultaneous integrated boost (DP‐SIB) VMAT technique, for a given patient. The PTV56, PTV63, PTV70, PTV78, and PTV84 are defined by the blue, violet, orange, pink, and yellow areas, respectively. The 95% prescription doses curves corresponding to the different PTVs are the thick lines with the blue, violet, orange, pink, and yellow‐green colours, respectively. The PTV coverages appear not to be different. However, BM collimator offers a slightly better dose sparing in the spinal cord (c) (red arrows) and in the brainstem (d) (orange arrows).

All the plans were normalized to deliver 95% of the prescribed dose (53.2 Gy) to 95% of the PTV56. Results were only obtained in this region. For all cases, satisfactory dose distributions for treatment delivery were obtained for each collimator. Typical dose distributions for a DP‐SIB case are shown in Fig. 1 for axial, sagittal and coronal views.

### A. Target volumes coverage

Table 1 provides mean and standard deviation values for PTVs volumes and specific values of DVH. For studied dose points, data dispersion was low. Indeed, maximum value of standard deviation was 6% of the mean dose.

For S‐SIB, Table 1 shows that Dmean of PTV56 for plans with MLCi2 were significantly higher (+0.5Gy) than those for plans with BM. Table 2 shows that HIs were significantly better with MLCi2 than with BM plans only for intermediate doses (63 Gy). Table 2 shows that CI was significantly better with BM plans than with MLCi2 plans for low and intermediate doses (56 Gy and 63 Gy).

For DP‐SIB, Table 1 shows that there were no significant differences on PTV doses between both collimators. Table 2 shows that all dosimetric indexes were very similar between MLCi2 plans and BM plans. Only CI value for the 56 Gy dose level was significantly better with BM plans.

**Table 1:** Dosimetric comparison for PTVs between 10 mm MLCI2 leaf width and 4 mm BM leaf width^a^

	*Standard SIB*			*Dose Painting SIB*		
	*MLCi2*	*BM*	$P^{b}$	*MLCi2*	*BM*	$P_{b}$
	$\text{PTV}56\;(301.3\pm 135.3\;{\text{cm}}^{3})$			$\text{PTV}56\;(304.3\pm 124.6\;{\text{cm}}^{3}$		
D2% (Gy)	61.5±2.1	60.6±2.2	0.01	59.7±1.0	60.4±1.7	0.09
D98% (Gy)	51.3±1.0	51.4±0.5	0.57	51.5±0.6	51.2±0.6	0.33
Dmean (Gy)	57.2±0.9	56.7±1.0	0.01	56.3±0.6	56.7±0.9	0.16
V95% (%)	95.0 (normalization point)			95.0 (normalization point)		
	$\text{PTV}63\;(382.3\pm 204.4\;{\text{cm}}^{3})$			$\text{PTV}63\;(413.5\pm 180.2\;{\text{cm}}^{3})$		
D2% (Gy)	69.1±1.5	68.7±2.0	0.11	68.7±1.3	68.9±1.1	0.45
D98% (Gy)	55.7±3.5	55.3±3.4	0.80	55.6±2.4	55.6±1.3	0.78
Dmean (Gy)	63.9±1.3	63.6±1.4	0.16	63.1±1.0	63.4±0.8	0.33
V95% (%)	93.6±6.8	90.1±5.3	0.06	88.7±5.7	89.7±3.7	0.48
	$\text{PTV}70\;(170.7\pm 101.8\;{\text{cm}}^{3})$			$\text{PTV}70\;(131.4\pm 74.8\;{\text{cm}}^{3})$		
D2% (Gy)	73.5±3.3	73.7±2.2	0.61	75.8±1.6	76.2±0.8	0.48
D98% (Gy)	65.9±1.5	65.6±1.3	0.28	65.3±1.4	65.8±1.3	0.26
Dmean (Gy)	70.8±1.1	70.3±1.5	0.10	70.5±0.9	70.8±0.9	0.16
V95% (%)	96.4±3.2	95.3±4.3	0.68	94.6±3.7	96.0±3.2	0.33
				$\text{PTV}78\;(18.4\pm 8.8\;{\text{cm}}^{3})$		
D2% (Gy)	N/A	N/A	N/A	80.9±0.9	81.2±0.8	0.48
D98% (Gy)	N/A	N/A	N/A	74.3±1.2	74.8±1.0	0.40
Dmean (Gy)	N/A	N/A	N/A	77.9±0.8	78.1±0.7	0.48
V95% (%)	N/A	N/A	N/A	98.0±1.7	98.8±1.1	0.16
				$\text{PTV}84\;(20.6\pm 9.4\;{\text{cm}}^{3})$		
D2% (Gy)	N/A	N/A	N/A	85.3±1.1	85.6±1.0	0.67
D98% (Gy)	N/A	N/A	N/A	79.3±1.0	79.7±0.8	0.26
Dmean (Gy)	N/A	N/A	N/A	82.7±1.0	83.1±1.0	0.21
V95% (%)	N/A	N/A	N/A	94.3±4.3	96.6±2.7	0.12

a Mean values and standard deviation; the PTVs of lower doses were defined by excluding the PTVs of higher doses.

b P‐value using Wilcoxon test.

**Table 2:** Comparison of dosimetric indexes between 10 mm MLCI2 leaf width and 4 mm BM leaf width^a^

	*Standard SIB*			*Dose Painting SIB*		
	*MLCi2*	*BM*	$p^{b}$	*MLCi2*	*BM*	$p^{b}$
		*56 Gy*			*56 Gy*	
HI	0.127±0.030	0.117±0.028	0.10	0.100±0.014	0.109±0.024	0.21
CI	1.363±0.079	1.299±0.058	0.00	1.411±0.103	1.327±0.038	0.04
COIN	0.690±0.229	0.623±0.258	0.28	0.339±0.124	0.353±0.111	0.16
		*63 Gy*			*63 Gy*	
HI	0.133±0.030	0.147±0.031	0.01	0.148±0.024	0.151±0.019	0.40
CI	1.320±0.505	1.209±0.302	0.03	1.130±0.123	1.141±0.110	0.89
COIN	0.975±0.568	0.880±0.359	0.08	0.488±0.097	0.541±0.107	0.36
		*70 Gy*			*70 Gy*	
HI	0.088±0.020	0.093±0.020	0.44	0.115±0.020	0.113±0.009	0.48
CI	1.604±0.520	1.442±0.432	0.16	1.390±0.236	1.479±0.276	0.16
COIN	0.577±0.535	0.624±0.389	0.07	0.614±0.114	0.565±0.144	0.07
					*78 Gy*	
HI	N/A	N/A		0.067±0.008	0.068±0.004	0.89
CI	N/A	N/A		1.550±0.254	1.561±0.255	1.00
COIN	N/A	N/A		0.635±0.079	0.631±0.079	1.00
					*84 Gy*	
HI	N/A	N/A		0.058±0.007	0.059±0.007	0.67
CI	N/A	N/A		1.177±0.200	1.232±0.150	0.40
COIN	N/A	N/A		0.766±0.064	0.958±0.050	0.78

a Mean values and standard deviation.

b P‐value using Wilcoxon test.

HI = Homogeneity Index (ideal values = 0); CI = Conformity Index (ideal values = 1); COIN = Conformal Index (ideal values = 1).

### B. Organs at risk sparing

#### B.1 Spinal cord

**Figure 2:**
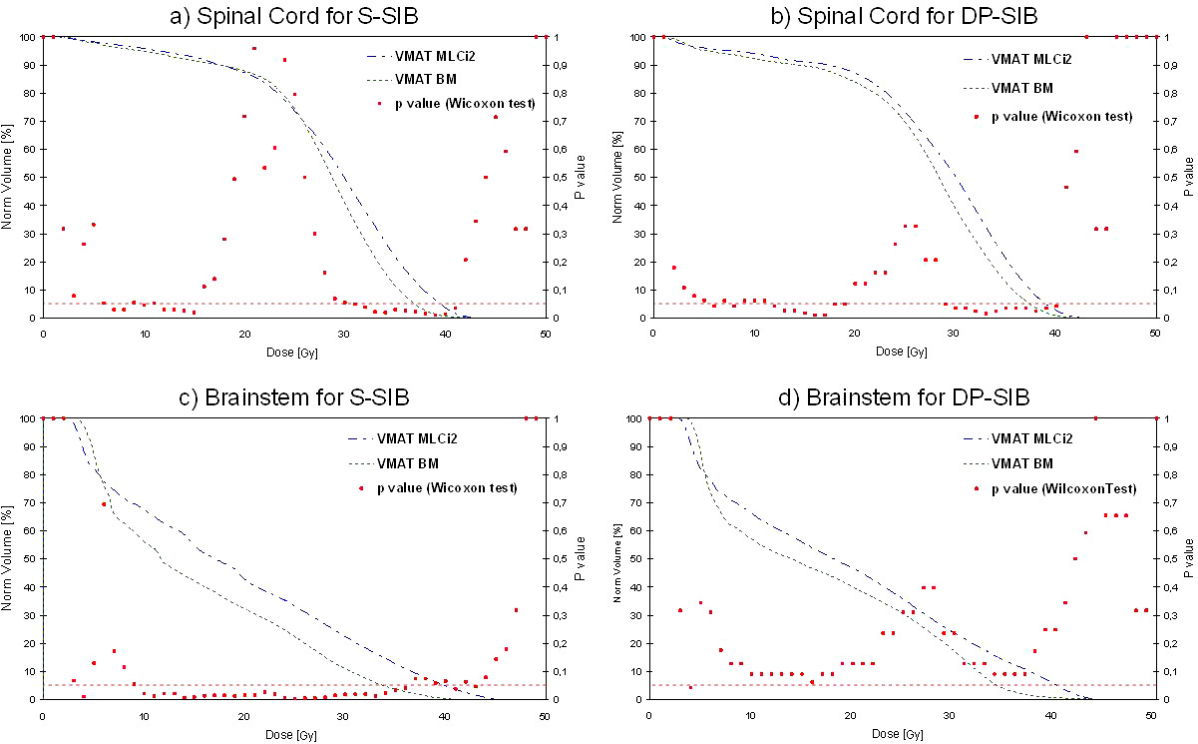
Spinal cord and brainstem DVHs for collimator leaf width (blue dashed lines = 10 mm MLCI2 leaf width; green lines = 4 mm BM leaf width) in VMAT technique: Figures (a) and (c) with a “standard” simultaneous integrated boost prescription (S‐SIB) (mean values for 16 patients); figures (b) and (d) with a dose painting (DP‐SIB) prescription (mean values for eight patients). Comparison test of the two curves has been made by the Wilcoxon test (p‐values are indicated with red points each Gy).

Figures 2(a) and 2(b) show that the spinal cord received lower doses with BM than with MLCi2 for S‐SIB and DP‐SIB techniques. The differences between the curves were more important for doses higher than 20 Gy and significant for doses comprised between 30 Gy and 40 Gy. Table 3 shows that D2% decreased significantly by 1.2 Gy with BM for S‐SIB technique, and Dmean decreased significantly by 1.6 Gy with BM for DP‐SIB technique.

**Table 3:** Dosimetric comparison for organ at risk between 10 mm MLCI2 leaf width and 4 mm BM leaf width^a^

	*Standard SIB*			*Dose Painting SIB*		
	*MLCi2*	*BM*	$p^{b}$	*MLCi2*	*BM*	$p^{b}$
	*Spinal Cord*			*Spinal Cord*		
D2% (Gy)	38.3±3.2	37.1±3.1	0.03	38.5±2.5	*Spinal Cord* 37.1±2.5	0.16
Dmean (Gy)	28.7±3.1	27.3±2.0	0.12	28.3±2.5	26.7±2.6	0.03
	*Brainstem*			*Brainstem*		
D2% (Gy)	37.2±6.6	33.0±7.1	0.04	34.1±14.2	31.3±13.2	0.06
Dmean (Gy)	18.7±6.4	15.2±4.7	0.00	16.8±8.3	14.6±7.1	0.09
	*Ipsi‐lateral Parotid*			*Ipsi‐lateral Parotid*		
Dmean (Gy)	39.5±10.8	40.9±12.0	0.47	44.0±10.7	44.2±10.2	0.33
Dmed (Gy)	39.3±15.7	38.7±16.3	0.44	44.1±13.9	45.2±14.2	0.16
V15Gy (%)	80.4±16.7	79.0±18.1	0.15	86.6±15.1	86.8±16.2	0.60
V30Gy (%)	62.7±21.2	63.5±23.3	0.84	70.9±22.0	70.8±21.6	0.58
V45Gy (%)	46.3±21.2	46.9±21.7	0.47	56.6±23.7	56.8±22.3	0.89
${\text{NTCP}}^{c}$	0.49±0.24	0.48±0.26	0.57	0.58±0.23	0.59±0.23	0.33
	*Controlateral Parotid*			*Controlateral Parotid*		
Dmean (Gy)	30.5±2.830.7±2.6	0.57	27.8±4.6	29.0±2.7	0.78
Dmed (Gy)	27.2±4.9	25.7±4.9	0.01	25.2±5.6	23.8±4.3	0.16
V15Gy (%)	66.9±5.4	67.3±8.9	0.20	68.0±11.6	69.6±13.9	0.16
V30Gy (%)	46.2±6.2	44.6±6.3	0.01	42.7±9.0	41.3±7.5	0.21
V45Gy (%)	29.3±6.8	28.5±6.5	0.11	26.3±8.2	26.1±7.3	0.67
NTCP**	0.28±0.06	0.27±0.05	0.13	0.25±0.06	0.25±0.05	0.67
	*Healthy Tissue*			*Healthy Tissue*		
V5Gy (%)	79.9±7.9	81.5±8.6	0.01	74.6±10.8	76.0±12.0	0.12
V10Gy (%)	64.9±7.9	63.4±8.0	0.01	59.9±8.3	58.6±9.4	0.26
V25Gy (%)	33.9±4.5	32.9±5.2	0.11	33.0±4.9	31.8±5.5	0.26
V50Gy (%)	5.9±1.5	5.0±1.4	0.00	6.0±1.1	5.0±1.1	0.04
	*Skin (3 mm superficial region)*			*Skin (3 mm superficial region)*		
Dmean (Gy)	13.8±2.113.7±2.4	0.12	*Skin* 14.0±1.5	13.8±1.9	0.40
V5Gy (%)	69.9±6.0	69.2±6.0	0.16	66.5±5.5	65.9±6.7	0.67
V10Gy (%)	49.0±6.0	48.0±7.2	0.15	47.8±4.6	46.5±6.7	0.26
V25Gy (%)	17.4±53.3	16.8±5.7	0.06	18.9±3.7	18.0±4.0	0.07
V50Gy (%)	2.0±1.3	2.4±1.8	0.12	2.5±1.2	2.9±1.3	0.12

a Mean values and standard deviation.

b P‐value using Wilcoxon test.

c NTCP: constants for parotid TD50 = 39.9 Gy, n = 1, m = 0.4 (defined by Dijkema et al.(^22^) and Houweling et al.(^23^)).

#### B.2 Brainstem

Figures 2(c) and 2(d) show that the brainstem received lower doses with BM than with MLCi2 for S‐SIB and DP‐SIB technique for doses higher than 7 Gy. Differences between the curves were only significant for S‐SIB technique for doses in the range of 10 Gy and 35 Gy. Table 3 shows that D2% and Dmean decreased significantly, by 4.2 Gy and 3.5 Gy respectively, with BM for S‐SIB technique. Figure 2(d) and Table 3 show no significant differences for DP‐SIB technique.

#### B.3 Parotids

Table 3 shows significant differences only for the controlateral parotid with S‐SIB technique in favor of BM. Dmed decreased significantly by 1.5 Gy with BM, and V30Gy decreased significantly from 46.2% with MLCi2 to 44.6% with BM.

#### B.4 Healthy tissue

For S‐SIB technique, Table 3 shows that V10Gy and V50Gy were significantly lower with BM than with MLCi2, but V5Gy was significantly lower with MLCi2. For DP‐SIB technique, only the differences on V50Gy were significant in favor of BM.

#### B.5 Skin

For S‐SIB and DP‐SIB techniques, Table 3 shows no significant differences between the two MLCs.

### C. Delivery efficiency

For S‐SIB and DP‐SIB techniques, Table 4 shows that the number of MU required with MLCi2 was 44% and 51% lower than those required with BM, respectively. To obtain an acceptable plan, two arcs were more frequently needed when using BM (12/16 for S‐SIB and 3/8 for DP‐SIB) than with MLCi2 (2/16 for S‐SIB and 7/8 for DP‐SIB). This difference was due to more complex fluence patterns.

**Table 4:** Comparison of efficiency between 10 mm MLCI2 leaf width and 4 mm BM leaf width

	*Standard SIB*			*Dose Painting SIB*		
	*MLCi2*	*BM*	$p^{a}$	*MLCi2*	*BM*	$p^{a}$
	*% of patients with 2 arcs*			*% of patients with 2 arcs*		
Percentage (%)	12.5	75	N/A	37	87.5	N/A
	*Number of Monitor Units*			*Number of Monitor Units*		
Mean values	537.8	774.8		548.2	829.5	
			0.00			0.01
Standard deviation	±51.3	±151.0		±44.9	±120.0	

a P‐value using Wilcoxon test.

## IV. DISCUSSION

The objective of this study was to evaluate the dosimetric impact of two MLCs with two different leaves width (4 mm vs. 10 mm) in “standard” SIB (16 patients) and “dose painting” SIB (eight patients) VMAT technique for head and neck prescriptions, which has been rarely explored in the literature. Due to the number of patients, general conclusions could be established only on S‐SIB group, and results obtained with DP‐SIB group can be used only to confirm the dosimetric impact of different leaf width in case of dose escalation.

We showed that MLC leaf width had no major impact on dosimetric indexes (Table 2) and on PTV doses (Table 1). The major impact of MLC leaf width on dose distribution was improvement of OAR sparing. Spinal cord and brainstem received fewer doses with BM in the two SIB prescriptions (D2% decreased by 1.3 Gy and 3.5 Gy) (Fig. 2). Such dose difference may have a clinical impact since it has been hypothesized in a recent randomized trial that fatigue during treatment could be related to the mean dose in the posterior fossa.^(24)^ Reducing the dose in such neurological structures may be also crucial in case of reirradiation.

To our knowledge, no previous study has investigated the influence of MLC leaf width on VMAT plans for HNC. Only one study, published by van Kesteren et al.,^(11)^ has investigated the impact of MLC, with 5 mm and 10 mm leaf width, for VMAT in case of prostate and rectum cancers. They found similar improvements of OARs sparing with thinner leaf width MLC; for both localizations, the mean doses of OARs decreased between 0.5 Gy and 2.5 Gy. Impact of MLC leaf width has been widely investigated for S&S and SW IMRT in different tumor localizations.^(^ ^10^ ^,^ ^25^, ^26^, ^27^ ^)^ For HNC with SIB prescription, two previous studies, published by Zwicker et al.^(9)^ and Yoganathan et al.,^(28)^ compared 5 mm and 10 mm MLC leaf width. The Zwicker study showed significant advantage to the use of 5 mm MLC leaf width rather than 10 mm MLC leaf width regarding target coverage and normal tissue sparing for S&S technique in case of Siemens MLCs. For Varian MLCs, the Yoganathan study found no major differences in terms of target coverage, OARs, and healthy tissue sparing between the 5 mm and 10 mm MLC leaf width. These findings do not fully agree between the two authors or with our own results. These differences could be explained by the use of different devices, MLCs, and TPS, and different delivery techniques. Wang et al.^(10)^ have investigated the influence of the two same Elekta MLCs in nasopharyngeal cases using S&S IMRT technique. They found different conclusions than ours; they showed that 4 mm MLC leaf width provided a better target coverage than 10 mm MLC leaf width, but no advantage on OAR sparing.

Regarding efficiency, we found that BM needed in average 44% and 51% more of MU than with MLCi2 for S‐SIB and DP‐SIB, respectively. Our results agree with those obtained by Burmeister et al.^(27)^ They found that the 10 mm leaf width plan required 40% fewer MUs in average than with the 5 mm leaf width plan for three tumor localizations (brain, pancreas, and prostate) in case of S&S IMRT technique. However Wang and colleagues found that MU decreased significantly (p=0.01) with 4 mm MLC leaf width (mean MU=698.2) than with 10 mm MLC leaf width (mean MU=745.7). These differences highlight the difficulty to provide general conclusions in this topic.

In order to better assess the impact of this leaf width related to geometrical configuration, we have created a numeric phantom with a C‐shaped tumor (external diameter=9.2cm) around a spherical OAR (external diameter=2.2cm),^(29)^ in which the gap between PTV and OAR was 0. 5 cm. The planning objective was to deliver 95% of the prescribed dose to 95% at the PTV volume, with a dose at the OAR being as low as possible. We have planned VMAT treatments using MLCi2 and BM. Regarding the PTV, the dose distribution was slightly more conformal with the MLCi2: CI value was 1.386 with MLCi2 and 1.426 with BM. The OAR sparing was better with BM: the mean dose to the OAR was 23.2% lower. At last, less monitor units were delivered using the MLCi2 (−13.4%). These results agree with our results obtained on complex HNC cases, in particular for dose distributions on the PTV. It could be explained because the phantom geometry was too simple compared to the complex real clinical HNC cases. Thus, to provide general conclusions available for specific anatomic sites, specific tumor site numerical phantoms should be created and investigated according to a consensus of scientific associations (AAPM, ESTRO). Currently, without such specific tests, it is still very important to investigate the impact of MLC for the specific equipment available in daily practice and for different tumors localizations.

In our study of BM and MLCi2, leaf width is the major MLC parameter influencing the dose distribution. However, other differences between both MLCs are MLC transmission, leaves interdigitation, and maximal leaf travel. MLC transmission is crucial particularly for low doses. MLCi2 and BM collimators have the same average MLC transmission (0.60%) measured under the leaves. But MLCi2 has two jaw pairs, which are parallel and perpendicular to the leaf direction. These jaws are movable during the dose delivery and allow decreasing the whole‐body dose. It is a possible explanation for the advantage of MLCi2 regarding the lowest dose received by the healthy tissues. For S‐SIB, V5Gy was 2% lower (p=0.01) with the MLCi2 than with the BM (Table 3). In our study, VMAT plans required more frequently two arcs with BM collimator than with MLCi2 collimator to reach an acceptable dose distribution. This could be explained by the more complex fluence patterns obtained with thinner leaf widths and by the leaf travel limitation of the BM. The field size limitation of the BM collimator required using two arcs to cover correctly the parts of PTVs that have largest dimensions in lateral direction. Therefore, the number of MUs increased respectively by 44% and by 51% with BM compared to MLCi2 for VMAT plans in standard and dose painting prescriptions (Table 4). This MU difference may have an impact on delivery time and integral dose and, therefore, possibly at the risk of second tumors.^(30)^

To investigate the influence of interdigitation, we have performed new plans with the interdigitation that has been enabled on MLCi2 (Interdig‐MLCi2) (results not provided here). We have compared the plans obtained with or without interdigitation for MLCi2 in case of DP‐SIB considered the more restrictive case. For PTVs, the differences on minimal; maximal and mean doses were inferior to ±1.0%. For spinal cord, maximal dose increased by 1.1% with Interdig‐MLCi2 than with MLCi2. For brainstem, maximal dose decreased by 2.4% with Interdig‐MLCi2. We found no dosimetric advantages to using MLCi2 interdigitation for HNC. Our results are in agreement with a previous study demonstrating that interdigitation of MLC leaves does not generate better plans using SmartArc algorithm.^(11)^ However, our study showed that interdigitation could improve efficiency; the mean number of MU decreased by 5% with Interdig‐MLCi2.

It must be pointed out that our results (likewise in other publications) are related to specific algorithms and planning strategies.^(^ ^4^ ^,^ ^31^ ^)^ In order to limit user dependency, Crijns et al.^(32)^ suggested integrating an automatic tool during optimization process. In our study, the optimization algorithm was always the same, the same physician always defined the volumes, and the same physicist always performed the planning. Pareto surface‐based techniques for multicriteria optimization is an new efficient method to minimize effect of planning strategies on plan quality. ^(33)^ Van Kesteren et al.^(11)^ have used Pareto fronts to investigated the impact of three MLCs on VMAT. For concaves PTVs (rectum cancer cases), they found dosimetric results that agree with our results — no major differences on dose conformity and improvement of OARs sparing.

## V. CONCLUSIONS

For complex HNC cases with standard and dose painting prescriptions, Beam Modulator (4 mm leaf width) and MLCi2 (10 mm leaf width) MLCs from Elekta provided satisfactory dose distributions for treatment delivery with VMAT technique. The major dosimetric advantage to using a narrow MLC leaf width is OAR sparing (spinal cord and brainstem). However, the delivery efficiency of VMAT plans was better with 10 mm leaf width in terms of monitor units and number of required arcs.

## Supporting information

Supplementary Material

Supplementary Material
