# Supplementary material for: Impact of MLC leaf width on volumetric‐modulated arc therapy planning for head and neck cancers
Source: J Appl Clin Med Phys. 2013 Nov 8;14(6):40–52. doi: 10.1120/jacmp.v14i6.4074 (PMC5714620; doi:10.1120/jacmp.v14i6.4074)
Supplement: Supplementary file 1 — Supplementary Material [file ACM2-14-040-s001.doc]

Interest

Caroline Lafond’ contribution is part of a PhD thesis supported by a grant from Elekta.

The authors alone are responsible for the content and writing of the paper.
