# Supplementary material for: Impact of MLC leaf width on volumetric‐modulated arc therapy planning for head and neck cancers
Source: J Appl Clin Med Phys. 2013 Nov 8;14(6):40–52. doi: 10.1120/jacmp.v14i6.4074 (PMC5714620; doi:10.1120/jacmp.v14i6.4074)
Supplement: Supplementary file 2 — Supplementary Material [file ACM2-14-040-s002.doc]

Impact of MLC leaf width on Volumetric Modulated Arc Therapy planning for head and neck cancers

**List all authors: first name, initial, last name:**

Caroline Lafond ^1, 2, 3^, Enrique Chajon ^1^, Anne Devillers ^1^, Guillaume Louvel ^1^, Sandra Toublanc ^1^, Mickael Olivier ^1^, Antoine Simon ^2,3^, Renaud de Crevoisier ^1, 2, 3^, Jean-Pierre Manens ^1, 2, 3^

**Department, Institution, City, State, Country:**

**^1^** *Centre Eugène Marquis, Rennes, France* 

**^2^** *INSERM U1099, Rennes, France*

**^3^** *Laboratoire du Traitement du Signal et de l’Image, University of Rennes 1, Rennes, France*

Corresponding author: Caroline Lafond

Corresponding author’s full mailing address:
*Centre Eugène Marquis
rue de la Bataille Flandres Dunkerque
CS 44229
35042 RENNES CEDEX
FRANCE
Tel.: (+33) 299253030
Fax: (+33) 299253033*
Email: c.lafond@rennes.unicancer.fr

Submitted: June 11, 2012

Accepted: June 26, 2013

Running title: MLC leaf width impact on VMAT

**Abstract:** This dosimetric study investigated the impact of multi-leaf collimators (MLC) leaf width in volumetric modulated arc therapy (VMAT) for head and neck cancers (HNC), either with a “standard” simultaneously integrated boost technique (S-SIB) or with a “dose-painting” SIB technique (DP-SIB). HNC patients were planned either with a S-SIB comprising 3 dose levels, from 56 to 70 Gy, (16 patients) or with a DP-SIB comprising 5 dose levels, from 56 to 84 Gy, (8 patients) in 35 fractions. Two VMAT plans were calculated for each SIB technique using two Elekta MLCs: MLCi2® (MLCi2) with 10 mm leaf width and Beam Modulator® (BM) with 4 mm leaf width. Dose distributions were evaluated by comparing doses on PTVs, main OARs and healthy tissue and by comparing conformation indexes. Treatment efficiencies were evaluated by comparing the number of monitor units and the number of needed arcs. Comparisons of the two MLCs depending on the two SIB techniques showed:

- Regarding PTVs: Dmean and D2% on lower doses PTV decreased respectively by 0.5 Gy (p=0.01) and 0.9 Gy (p=0.01) with BM than with MLCi2 for S-SIB; no significant difference was found for DP-SIB;

- Regarding OARs: For spinal cord and brainstem, D2% decreased respectively by 1.2 Gy (p=0.03) and 4.2 Gy (p=0.04) with BM than with MLCi2 for S-SIB; for contro-lateral parotid D50% decreased by 1.5 Gy (p=0.01) with BM than with MLCi2 for S-SIB;

- Regarding treatment efficiency: the number of monitor units was 44% (p=0.00) and 51% (p=0.01) higher with BM for S-SIB and DP-SIB respectively. Two arcs were more frequently needed with BM to reach an acceptable dose distribution.

This study demonstrated that Beam Modulator (4 mm leaf width) and MLCi2 (10 mm leaf width) MLCs from Elekta provided satisfactory dose distributions for treatment delivery with VMAT technique for complex HNC cases with standard and dose painting prescriptions. OAR sparing was better with BM, mainly for brainstem and spinal cord. However, delivery efficiency of VMAT plans was better with MLCi2.

PACS number: 87.56.N-, 87.56.nk, 87.55.D-

Keywords: VMAT; Dosimetry; Head and neck cancer; Multileaf collimators; Leaf width

**I. Introduction**

Radiotherapy is one of the crucial components of the modern multidisciplinary approach of head and neck cancer (HNC) treatment. Intensity-modulated radiotherapy (IMRT) provides the benefit of delivering conformal doses to the target volume while maintaining low doses to the critical organs (1).

Otto has recently introduced Volumetric Modulated Arc Therapy (VMAT) technique (2). VMAT is a natural progression of IMRT delivery, combining the possibility to vary simultaneously the dose rate, the speed of gantry rotation and the multileaf collimator (MLC) shape. Several studies showed that VMAT technique can achieve at least equivalent and possibly superior dose distributions than Step and Shoot (S&S) or Sliding Widow (SW) IMRT. Moreover, efficiency of the treatment is often improved with the VMAT technique by decreasing significantly the number of Monitor Units (MU) and delivery time (3-8).

A few studies investigated the impact of MLC leaf width on IMRT techniques for HNC. Zwicker et al. have proved the advantage to use narrower MLC leaf width for HNC in S&S IMRT (9). Wang et al. have investigated the impact of MLC leaf width for nasopharyngeal cancer in S&S technique (10). They showed that narrower MLC leaf width only improved dose coverage and treatment efficiency. No previous study investigated the impact of MLC leaf width on VMAT technique for HNC using specifically simultaneous integrated boost (SIB) prescriptions. Only one recent study compared two MLC leaf width in VMAT for prostate and rectum cancer with a favorable effect when using more narrow MLC leaf width (11).

For HNC, IMRT could be used to increase local control by escalating the dose in the most radio-resistant or proliferative part of the tumor (12). Technical feasibility of 18F-fluorodesoxyglucose positons emission tomography (FDG-PET) imaging dose painting has been recently shown by different groups (13;14). Dose levels are usually arbitrarily determined. Madani et al. (15) shown, in a phase I dose escalation trial, the feasibility of delivering two dose levels: 72,5 Gy (78,2 Gy NID2Gy [normalized isoeffective dose]) and 77,5 Gy (86,7 Gy NID2Gy). Following this principle, we explored the influence of multi-leaf collimator in a theoretical scenario of dose painting requiring multiple target sub-volumes defined by FDG-PET.

The aim of this study was to investigate the impact of MLC leaf width on VMAT dose distribution for head and neck cancer (HNC) using two different SIB prescriptions. The first prescription, considered as “standard”, comprised three dose levels (S-SIB). The second one, considered as a more “dose-painting” approach, comprised five dose levels (DP-SIB).

**II. Materials and methods**

**A. Patients**

CT scans of 16 locally advanced non-metastatic HNC cases treated by S&S IMRT with curative intention were selected for this plan comparison study. The primary site was the oropharynx in 4 cases, the hypopharynx in 8, the oral cavity in 3 and the larynx in one case. The American Joint Committee on Cancer (AJCC) staging system was used according to the primary tumor location. The T stage was T1 in one, T2 in 3, T3 in 5, and T4 in 7 cases. The N stage was N0 in 6, N1 in 2, N2 in 5, and N3 in 3 cases.

All patients were immobilized by using a custom Aquaplast masks holding both neck and shoulders. Computed tomography contrast-enhanced images indexed every 3 mm were acquired extending from the vertex to the carina. All target volumes and OAR were delineated slice by slice on CT images. The definition of target volumes was made in accordance with the ICRU Report 83 (16). The international guidelines for HNC cancer were used to define clinical target volumes (CTV) according to the site of the primary tumor (17). In brief, the gross target volume (GTV) was defined by clinical examination and computed tomography, FDG-PET. The CTVs were defined by the GTV plus areas considered containing potential microscopic disease or by the lymph node levels at risk of subclinical disease. The planning target volume (PTV), aimed to account for setup uncertainties, was defined using an additional margin of 5 mm around the CTV.

For 8 patients, PET imaging was acquired in the same position than the planning CT scan 60 min after injection of 4 MBq/Kg of FDG. Two metabolic tumor volumes (MTVs) were delineated on a workstation (General Electric, Advantage windows) using thresholds of 40% and 70% of the maximum tumor uptake, called 40%-MTV and 70%-MTV respectively. Both MTVs were transferred to the Treatment Planning System (TPS).

**B. Planning objectives**

All treatments were planned using a SIB technique.

For S-SIB technique, the three PTVs were defined as follows: PTV70 encompassed the gross tumor plus a margin of 5 mm; PTV63 was defined as the high-risk sub-clinical disease volume plus a 5 mm margin and by excluding the PTV70; PTV56 was defined as the low-risk subclinical disease plus a 5 mm margin and by excluding the PTV70 and the PTV63. Each volume was typically treated once daily, five days a week, in 35 fractions (PTV70: 2 Gy/fr for a total dose of 70 Gy; PTV63: 1.8 Gy/fr for a total dose of 63 Gy; PTV56: 1.6 Gy/fr for a total dose of 56 Gy).

For DP-SIB technique, the two additional PTVs were defined as follows: PTV 84 Gy encompassed the 70%-MTV plus a margin of 5 mm and PTV 78 Gy encompassed the 40%-MTV plus a margin of 5 mm and by avoiding the PTV 84 Gy. In case of DP-SIB, PTV 70 Gy was modified to avoid the PTV 84 Gy and the PTV 78 Gy. The prescribed doses for those subvolumes were 84 Gy (2,4 Gy/fr) and 78 Gy (2,23 Gy/fr) in 35 fractions.

The target dose objectives were: more than 95% of any PTV should receive more than 95 % of the prescribed dose or more than 98% of any PTV should receive more than 90% of the prescribed dose, no more than 20% of any PTV could receive more than 110% of the prescribed dose, and no more than 1% or 1 cm^3^ of the tissue outside the PTV should receive more than 110% of the dose prescribed to the primary dose target (PTV 70 Gy). To accept a plan, the rules were: at least two thirds of the PTVs had to respect the objectives V95% ≥95% and at least two thirds of the PTVs had to respect the objectives V90% ≥98%. For inverse planning and evaluation dose, target volumes were limited to avoid the first 3 mm of the build-up region (18).

The following OAR dose constraints were used: the brainstem and spinal cord maximal doses were 50 Gy and 45 Gy respectively. Planning objectives were: mean dose < 26 Gy or the maximal dose received by 50% of the volume < 30 Gy for the parotid glands.

**C. Collimators specification**

The plans were calculated for two Elekta internal collimators: MLCi2® (MLCi2) and Beam modulator® (BM).

The MLCi2 has 40 pairs of leaves with a 10 mm width at the isocenter. The minimum opposite leaf gap is 0.5 cm and the maximum field size is 40×40 cm×cm. The maximum distance between leaves on the same leaf guide is 32.5 cm (12.5 cm over central axis). MLCi2 was used without leaves interdigitation. Under the leaves there are auto tracking backup diaphragms which are movable during the treatment for automated leakage reduction. Perpendicular jaws are also movable during the treatment. The average MLC transmission is 0.60% measured under the leaves and 0.13% measured under the leaves and parallel jaws. The leaves have a rounded leaf end and a flat side.

The BM has 40 pairs of leaves with a width at the isocenter of 4 mm. The minimum opposite leaf gap is 0.05 cm and the maximum field size is 21×16 cm×cm. The maximum distance between leaves on the same leaf guide is 21 cm (full field travel) and leaves interdigitation is allowed. The average MLC transmission is 0.60% measured under the leaves. There are no additional jaws. The leaves have a rounded leaf end and a flat side.

**D. VMAT plan**

All plans were planned with the Philips TPS, Pinnacle (version 9.0), using a 6 MV photons beams from an Elekta Synergy linear accelerator. Final dose was computed with a collapsed cone algorithm. To achieve the desired dose distribution, VMAT plans used either one full arc in clock-wise direction or two full arcs in clock- and counter-clock wise directions in a same plane according to the rules that have been explained in the “planning objectives” section. Plans were optimized with the SmartArc algorithm (19). For each patient and each MLC, inverse constraints were optimized. Each arc was made up of 90 control points (CP) separated by 4°. For BM, the collimator angle was set at 90° to have the larger maximal dimension of the MLC (i.e. 21 cm) in the cranio-caudal direction. MLC positions, MU and dose rate were optimized for each CP. During the delivery, the MLC moved while the gantry rotated at a variable MU/degree. To deliver a variable MU/degree, the treatment control system (TCS) from the linear accelerator optimized the gantry speed and the dose rate to minimize the beam hold-offs. The leaves of the MLC and the gantry moved linearly between two CP, leaves and gantry speeds were optimized to get the faster delivery. All linear and rotational movements during radiation had to respect the minimum variations of 0.3 MU/cm and 0.1 MU/degree respectively. For our accelerator the minimum and maximum dose rate allowed are 25 MU/min and 400 MU/min respectively. The maximum gantry speed is 6 degree/s.

**E. Evaluation tools**

Mean Dose-Volume Histograms (DVHs) were computed for PTVs and main OARs. Some specific values of DVH were analyzed for the 16 patients. Considering PTV, maximum dose (D2%), minimum dose (D98%), mean dose (Dmean) and V95% were analyzed. For spinal cord and brainstem, D2% and Dmean were analyzed. For ipsi- and contro-lateral parotids, Dmean, median dose (Dmed), V15Gy, V30Gy and V45Gy were analyzed. For healthy tissues, defined as external contours minus PTVs, we have analyzed V5Gy, V10Gy, V25Gy and V50Gy. For skin, defined as superficial region with a 3 mm thickness, Dmean, V5Gy, V10Gy, V25Gy and V50Gy were analyzed. The target homogeneity was expressed by the Homogeneity Index (HI) defined as (D5%-D95%)/Dmean. The degree of conformity was measured with the Conformity Index CI defined as the ratio between the reference isodose (95% of the prescribed dose) volume and the volume of the PTV (20). COnformal INdex (COIN) as defined by Baltas (21) was also calculated. NTCP values were calculated for parotids with the parameters defined by Dijkema et al. : TD50=39.9 Gy, n=1, m=0.4 (22;23).

To evaluate the dose delivery efficiency, the numbers of Monitor Units (MU) were compared.

Statistical analysis used two-sided Wilcoxon-signed rank test, a nonparametric test, calculated with the IBM software, PASW (version 18.0.0). A value of p<0.05 was considered statistically significant.

**III. Results**

All the plans were normalized to deliver 95% of the prescribed dose (53.2 Gy) to 95% of the PTV56. Results were only obtained in this region. For all cases, satisfactory dose distributions for treatment delivery were obtained for each collimator. Typical dose distributions for a DP-SIB case are shown in Figure 1 for axial, sagittal and coronal views.

**A. Target volumes coverage**

Table 1 provides mean and standard deviation values for PTVs volumes and specific values of DVH. For studied dose points, data dispersion was low. Indeed maximum value of standard deviation was 6% of the mean dose.

For S-SIB, Table 1 shows that Dmeans of PTV56 for plans with MLCi2 were significantly higher (+ 0.5 Gy) than those for plans with BM. Table 2 shows that HIs were significantly better with MLCi2 than with BM plans only for intermediate doses (63 Gy). Table 2 shows that CI was significantly better with BM plans than with MLCi2 plans for low and intermediate doses (56 Gy and 63 Gy).

For DP-SIB, Table 1 shows that there were no significant differences on PTV doses between both collimators. Table 2 shows that all dosimetric indexes were very similar between MLCi2 plans and BM plans. Only CI value for the 56 Gy dose level was significantly better with BM plans.

**B. Organs at risk sparing**

***B.1 Spinal cord***

Figure 2a and Figure 2b show that the spinal cord received lower doses with BM than with MLCi2 for S-SIB and DP-SIB techniques. The differences between the curves were more important for doses higher than 20 Gy and significant for doses comprised between 30 Gy and 40 Gy. Table 3 shows that D2% decreased significantly by 1.2 Gy with BM for S-SIB technique, and Dmean decreased significantly by 1.6 Gy with BM for DP-SIB technique.

***B.2 Brainstem***

Figure 2c and Figure 2d show that the brainstem received lower doses with BM than with MLCi2 for S-SIB and DP-SIB technique for doses higher than 7 Gy. Differences between the curves were only significant for S-SIB technique for doses in the range of 10 Gy and 35 Gy. Table 3 shows that D2% and Dmean decreased significantly, by 4.2 Gy and 3.5 Gy respectively, with BM for S-SIB technique. Figure 2d and Table 3 show no significant differences for DP-SIB technique.

***B.3 Parotids***

Table 3 shows significant differences only for the contro-lateral parotid with S-SIB technique in favor of BM. Dmed decreased significantly by 1.5 Gy with BM and V30Gy decreased significantly from 46.2% with MLCi2 to 44.6% with BM.

***B.4 Healthy Tissue***

For S-SIB technique, Table 3 shows that V10Gy and V50Gy were significantly lower with BM than with MLCi2 but V5Gy was significantly lower with MLCi2. For DP-SIB technique, only the differences on V50Gy were significant in favor of BM.

***B.5 Skin***

For S-SIB and DP-SIB techniques, Table 3 shows no significant differences between the two MLCs.

**C. Delivery efficiency**

For S-SIB and DP-SIB techniques, Table 4 shows that the number of MU required with MLCi2 was 44% and 51% lower than those required with BM respectively. To obtain an acceptable plan, two arcs were more frequently needed when using BM (12/16 for S-SIB and 3/8 for DP-SIB) than MLCi2 (2/16 for S-SIB and 7/8 for DP-SIB). This difference was due to more complex fluence patterns.

**IV. Discussion**

The objective of this study was to evaluate the dosimetric impact of two MLCs with two different leaves width (4 mm versus 10 mm) in “standard” SIB (16 patients) and “dose painting” SIB (8 patients) VMAT technique for head and neck prescriptions, which has been rarely explored in the literature. Due to the number of patients, general conclusions could be established only on S-SIB group and results obtained with DP-SIB group can be used only to confirm the dosimetric impact of different leaf width in case of dose escalation.

We showed that MLC leaf width had no major impact on dosimetric indexes (Table 2) and on PTV doses (Table 1). The major impact of MLC leaf width on dose distribution was improvement of OAR sparing. Spinal cord and brainstem received fewer doses with BM, in the two SIB prescriptions (D2% decreased by 1.3 Gy and 3.5 Gy) (Figure 2). Such dose difference may have a clinical impact since it has been hypothesized in a recent randomized trial that fatigue during treatment could be related to the mean dose in the posterior fossa (24). Reducing the dose in such neurological structures may be also crucial in case of re-irradiation.

To our knowledge, no previous study has investigated the influence of MLC leaf width on VMAT plans for HNC. Only one study, published by van Kesteren et al., has investigated the impact of MLC, with 5 mm and 10 mm leaf width, for VMAT in case of prostate and rectum cancers (11). They found similar improvements of OARs sparing with thinner leaf width MLC; for both localizations, the mean doses of OARs decreased between 0.5 Gy and 2.5 Gy. Impact of MLC leaf width has been widely investigated for S&S and SW IMRT in different tumor localizations (10;25-27). For HNC with SIB prescription, two previous studies, published by Zwicker et al. and Yoganathan et al., compared 5 mm and 10 mm MLC leaf width (9;28). Zwicker et al. showed significant advantage to use 5 mm MLC leaf width than 10 mm MLC leaf width regarding target coverage and normal tissue sparing for S&S technique in case of Siemens MLCs (9). For Varian MLCs, Yoganathan et al found no major differences in terms of target coverage, OAR and healthy tissue sparing between the 5 mm and 10 mm MLC leaf width (28). These findings do not fully agree between the two authors or with our own results. These differences could be explained by the use of different devices, MLCs and TPS, and different delivery techniques. Wang et al., has investigated the influence of the two same Elekta MLCs in nasopharyngeal cases using S&S IMRT technique (10). They found different conclusions than ours; they showed that 4 mm MLC leaf width provided a better target coverage than 10 mm MLC leaf width but no advantage on OAR sparing. Regarding efficiency we found that BM needed in average 44% and 51% more of MU than with MLCi2 for S-SIB and DP-SIB respectively. Our results agree with those obtained by Burmeister et al (27). They found that the 10 mm leaf width plan required 40 % fewer MU in average than with the 5 mm leaf width plan for three tumor localizations (brain, pancreas and prostate) in case of S&S IMRT technique. However Wang et al found that MU decreased significantly (p <0.01) with 4 mm MLC leaf width (mean MU = 698.2 MU) than with 10 mm MLC leaf width (mean MU = 745.7 MU). These differences highlight the difficulty to provide general conclusions in this topic. In order to better assess the impact of this leaf width related to geometrical configuration, we have created a numeric phantom with a C shaped tumor (external diameter = 9.2 cm) around a spherical OAR (external diameter = 2.2 cm) (29), the gap between PTV and OAR was 0.5 cm. The planning objective was to deliver 95% of the prescribed dose to 95% at the PTV volume, with a dose at the OAR being as low as possible. We have planned VMAT treatments using MLCi2 and BM. Regarding the PTV, the dose distribution was slightly more conformal with the MLCi2: CI value was 1.386 with MLCi2 and 1.426 with BM. The OAR sparing was better with BM: the mean dose to the OAR was 23.2% lower. At last, less monitor units were delivered using the MLCi2 (-13.4%). These results agree with our results obtained on complex HNC cases, in particular for dose distributions on the PTV. It could be explained because the phantom geometry was too simple compared to the complex real clinical HNC cases. Thus, to provide general conclusions available for specific anatomic sites, specific tumor site numerical phantoms should be created and investigated according to a consensus of scientific associations (AAPM, ESTRO…). Currently, without such specific tests, it is still very important to investigate the impact of MLC for the specific equipment available in daily practice and for different tumors localizations.

In our study of BM and MLCi2, leaf width is the major MLC parameter influencing the dose distribution. However, other differences between both MLC are MLC transmission, leaves interdigitation and maximal leaves travel. MLC transmission is crucial particularly for low doses. MLCi2 and BM collimators have the same average MLC transmission (0.60%) measured under the leaves. But MLCi2 has two jaws pairs, which are parallel and perpendicular to the leaf direction. These jaws are movable during the dose delivery and allow decreasing the whole-body dose. It is a possible explanation for the advantage of MLCi2 regarding the lowest dose received by the healthy tissues. For S-SIB, V5Gy was 2% lower (p=0.01) with the MLCi2 than with the BM (Table 3). In our study VMAT plans required more frequently two arcs with BM collimator than with MLCi2 collimator to reach an acceptable dose distribution. This could be explained by the more complex fluence patterns obtained with thinner leaf widths and by the leaf travel limitation of the BM. The field size limitation of the BM collimator required using two arcs to cover correctly the parts of PTVs that have largest dimensions in lateral direction. Therefore, the number of MU increased respectively by 44% and by 51% with BM compared to MLCi2 for VMAT plans in standard and dose painting prescriptions (Table 4). This MU difference may have an impact on delivery time and integral dose and therefore possibly on the risk of second tumors (30). To investigate the influence of interdigitation, we have performed new plans with the interdigitation that has been enabled on MLCi2 (Interdig-MLCi2) (results not provided here). We have compared the plans obtained with or without interdigitation for MLCi2 in case of DP-SIB considered the more restrictive case. For PTVs, the differences on minimal, maximal and mean doses were inferior to ± 1.0 %. For spinal cord, maximal dose increased by 1.1 % with Interdig-MLCi2 than with MLCi2. For brainstem, maximal dose decreased by 2.4 % with Interdig-MLCi2. We found no dosimetric advantages to use MLCi2 interdigitation for HNC. Our results are in agreement with a previous study demonstrating that interdigitation of MLC leaves does not generate better plans using SmartArc algorithm (11). However our study showed that interdigitation could improve efficiency: the mean number of MU decreased by 5% with Interdig-MLCi2.

It must be pointed out that our results (likewise in other publications) are related to specific algorithms and planning strategies (4;31). In order to limit user dependency, Crijns et al. suggested to integrate an automatic tool during optimization process (32). In our study, the optimization algorithm was always the same, the same physician always defined the volumes and the same physicist always performed the planning. Pareto surface-based techniques for multicriteria optimization is an new efficient method to minimize effect of planning strategies on plan quality (33). Van Kesteren et al. have used Pareto fronts to investigated the impact of three MLCs on VMAT (11). For concaves PTVs (rectum cancer cases), they found dosimetric results that agree with our results: no major differences on dose conformity and improvement of OARs sparing.

**V. Conclusion**

For complex HNC cases with standard and dose painting prescriptions, BeamModulator (4 mm leaf width) and MLCi2 (10 mm leaf width) MLCs from Elekta provided satisfactory dose distributions for treatment delivery with VMAT technique. The major dosimetric advantage to use a narrow MLC leaf width is OAR sparing (spinal cord and brainstem). However, the delivery efficiency of VMAT plans was better with 10 mm leaf width in terms of monitor units and number of required arcs.

**Declaration of interest:**

Caroline Lafond’ contribution is part of a PhD thesis supported by a grant from Elekta.

The authors alone are responsible for the content and writing of the paper.

**REFERENCES**

1. Intensity-modulated radiotherapy: current status and issues of interest. Int.J Radiat.Oncol Biol.Phys. 2001 Nov 15;51(4):880-914.

2. Otto K. Volumetric modulated arc therapy: IMRT in a single gantry arc. Med.Phys. 2008 Jan;35(1):310-7.

3. Bertelsen A, Hansen CR, Johansen J, Brink C. Single Arc Volumetric Modulated Arc Therapy of head and neck cancer. Radiother.Oncol. 2010 Feb 24;95(2):142-8.

4. Vanetti E, Clivio A, Nicolini G, Fogliata A, Ghosh-Laskar S, Agarwal JP, Upreti RR, Budrukkar A, Murthy V, Deshpande DD, et al. Volumetric modulated arc radiotherapy for carcinomas of the oro-pharynx, hypo-pharynx and larynx: a treatment planning comparison with fixed field IMRT. Radiother.Oncol. 2009 Jul;92(1):111-7.

5. Guckenberger M, Richter A, Krieger T, Wilbert J, Baier K, Flentje M. Is a single arc sufficient in volumetric-modulated arc therapy (VMAT) for complex-shaped target volumes? Radiother.Oncol. 2009 Sep 10;93(2):259-65.

6. Verbakel WF, Cuijpers JP, Hoffmans D, Bieker M, Slotman BJ, Senan S. Volumetric intensity-modulated arc therapy vs. conventional IMRT in head-and-neck cancer: a comparative planning and dosimetric study. Int.J.Radiat.Oncol.Biol.Phys. 2009 May 1;74(1):252-9.

7. Kjaer-Kristoffersen F, Ohlhues L, Medin J, Korreman S. RapidArc volumetric modulated therapy planning for prostate cancer patients. Acta Oncol. 2009;48(2):227-32.

8. Bedford JL, Nordmark H, V, McNair HA, Aitken AH, Brock JE, Warrington AP, Brada M. Treatment of lung cancer using volumetric modulated arc therapy and image guidance: a case study. Acta Oncol. 2008;47(7):1438-43.

9. Zwicker F, Hauswald H, Nill S, Rhein B, Thieke C, Roeder F, Timke C, Zabel-du BA, Debus J, Huber PE. New multileaf collimator with a leaf width of 5 mm improves plan quality compared to 10 mm in step-and-shoot IMRT of HNC using integrated boost procedure. Strahlenther.Onkol. 2010 Jun;186(6):334-43.

10. Wang S, Gong Y, Xu Q, Bai S, Lu Y, Jiang Q, Chen N. Impacts of multileaf collimators leaf width on intensity-modulated radiotherapy planning for nasopharyngeal carcinoma: analysis of two commercial Elekta devices. Med.Dosim. 2010 May 18;36(2):153-9.

11. van KZ, Janssen TM, Damen E, van Vliet-Vroegindeweij C. The dosimetric impact of leaf interdigitation and leaf width on VMAT treatment planning in Pinnacle: comparing Pareto fronts. Phys.Med Biol. 2012 Apr;%20;57(10):2943-52.

12. Toma-Dasu I, Dasu A, Brahme A. Dose prescription and optimisation based on tumour hypoxia. Acta Oncol. 2009;48(8):1181-92.

13. Vanderstraeten B, Duthoy W, De GW, De NW, Thierens H. [18F]fluoro-deoxy-glucose positron emission tomography ([18F]FDG-PET) voxel intensity-based intensity-modulated radiation therapy (IMRT) for head and neck cancer. Radiother.Oncol 2006 Jun;79(3):249-58.

14. Thorwarth D, Eschmann SM, Paulsen F, Alber M. Hypoxia dose painting by numbers: a planning study. Int.J Radiat.Oncol Biol.Phys. 2007 May 1;68(1):291-300.

15. Madani I, Duthoy W, Derie C, De GW, Boterberg T, Saerens M, Jacobs F, Gregoire V, Lonneux M, Vakaet L, et al. Positron emission tomography-guided, focal-dose escalation using intensity-modulated radiotherapy for head and neck cancer. Int.J Radiat.Oncol Biol.Phys. 2007 May 1;68(1):126-35.

16. Journal of the ICRU. Vol. 10, 2010.

17. Gregoire V, Levendag P, Ang KK, Bernier J, Braaksma M, Budach V, Chao C, Coche E, Cooper JS, Cosnard G, et al. CT-based delineation of lymph node levels and related CTVs in the node-negative neck: DAHANCA, EORTC, GORTEC, NCIC,RTOG consensus guidelines. Radiother.Oncol 2003 Dec;69(3):227-36.

18. Thomas SJ, Hoole AC. The effect of optimization on surface dose in intensity modulated radiotherapy (IMRT). Phys.Med.Biol. 2004 Nov 7;49(21):4919-28.

19. Bzdusek K, Friberger H, Eriksson K, Hardemark B, Robinson D, Kaus M. Development and evaluation of an efficient approach to volumetric arc therapy planning. Med.Phys. 2009 Jun;36(6):2328-39.

20. Huchet A, Caudry M, Belkacemi Y, Trouette R, Vendrely V, Causse N, Recaldini L, Atlan D, Maire JP. [Volume-effect and radiotherapy [II]. Part II: volume-effect and normal tissue]. Cancer Radiother. 2003 Oct;7(5):353-62.

21. Baltas D, Kolotas C, Geramani K, Mould RF, Ioannidis G, Kekchidi M, Zamboglou N. A conformal index (COIN) to evaluate implant quality and dose specification in brachytherapy. Int.J.Radiat.Oncol.Biol.Phys. 1998 Jan 15;40(2):515-24.

22. Dijkema T, Raaijmakers CP, Ten Haken RK, Roesink JM, Braam PM, Houweling AC, Moerland MA, Eisbruch A, Terhaard CH. Parotid gland function after radiotherapy: the combined michigan and utrecht experience. Int.J Radiat.Oncol Biol.Phys. 2010 Oct 1;78(2):449-53.

23. Houweling AC, Philippens ME, Dijkema T, Roesink JM, Terhaard CH, Schilstra C, Ten Haken RK, Eisbruch A, Raaijmakers CP. A comparison of dose-response models for the parotid gland in a large group of head-and-neck cancer patients. Int.J Radiat.Oncol Biol.Phys. 2010 Mar 15;76(4):1259-65.

24. Nutting CM, Morden JP, Harrington KJ, Urbano TG, Bhide SA, Clark C, Miles EA, Miah AB, Newbold K, Tanay M, et al. Parotid-sparing intensity modulated versus conventional radiotherapy in head and neck cancer (PARSPORT): a phase 3 multicentre randomised controlled trial. Lancet Oncol. 2011 Feb;12(2):127-36.

25. Jacob V, Bayer W, Astner ST, Busch R, Kneschaurek P. A Planning Comparison of Dynamic IMRT for Different Collimator Leaf Thicknesses with Helical Tomotherapy and RapidArc for Prostate and Head and Neck Tumors. Strahlenther.Onkol. 2010 Aug 30;186(9):502-10.

26. Nill S, Tucking T, Munter MW, Oelfke U. Intensity modulated radiation therapy with multileaf collimators of different leaf widths: a comparison of achievable dose distributions. Radiother.Oncol. 2005 Apr;75(1):106-11.

27. Burmeister J, McDermott PN, Bossenberger T, Ben-Josef E, Levin K, Forman JD. Effect of MLC leaf width on the planning and delivery of SMLC IMRT using the CORVUS inverse treatment planning system. Med.Phys. 2004 Dec;31(12):3187-93.

28. Yoganathan SA, Mani KR, Das KJ, Agarwal A, Kumar S. Dosimetric effect of multileaf collimator leaf width in intensity-modulated radiotherapy delivery techniques for small- and large-volume targets. J Med.Phys. 2011 Apr;36(2):72-7.

29. Ezzell GA, Burmeister JW, Dogan N, LoSasso TJ, Mechalakos JG, Mihailidis D, Molineu A, Palta JR, Ramsey CR, Salter BJ, et al. IMRT commissioning: multiple institution planning and dosimetry comparisons, a report from AAPM Task Group 119. Med.Phys. 2009 Nov;36(11):5359-73.

30. Verellen D, Vanhavere F. Risk assessment of radiation-induced malignancies based on whole-body equivalent dose estimates for IMRT treatment in the head and neck region. Radiother.Oncol. 1999 Dec;53(3):199-203.

31. Cozzi L, Dinshaw KA, Shrivastava SK, Mahantshetty U, Engineer R, Deshpande DD, Jamema SV, Vanetti E, Clivio A, Nicolini G, et al. A treatment planning study comparing volumetric arc modulation with RapidArc and fixed field IMRT for cervix uteri radiotherapy. Radiother.Oncol. 2008 Nov;89(2):180-91.

32. Crijns W, Budiharto T, Defraene G, Verstraete J, Depuydt T, Haustermans K, Van den HF. IMRT-based optimization approaches for volumetric modulated single arc radiotherapy planning. Radiother.Oncol. 2010 Feb 18;95(2):149-52.

33. Craft DL, Hong TS, Shih HA, Bortfeld TR. Improved planning time and plan quality through multicriteria optimization for intensity-modulated radiotherapy. Int.J Radiat.Oncol Biol.Phys. 2012 Jan 1;82(1):e83-e90.


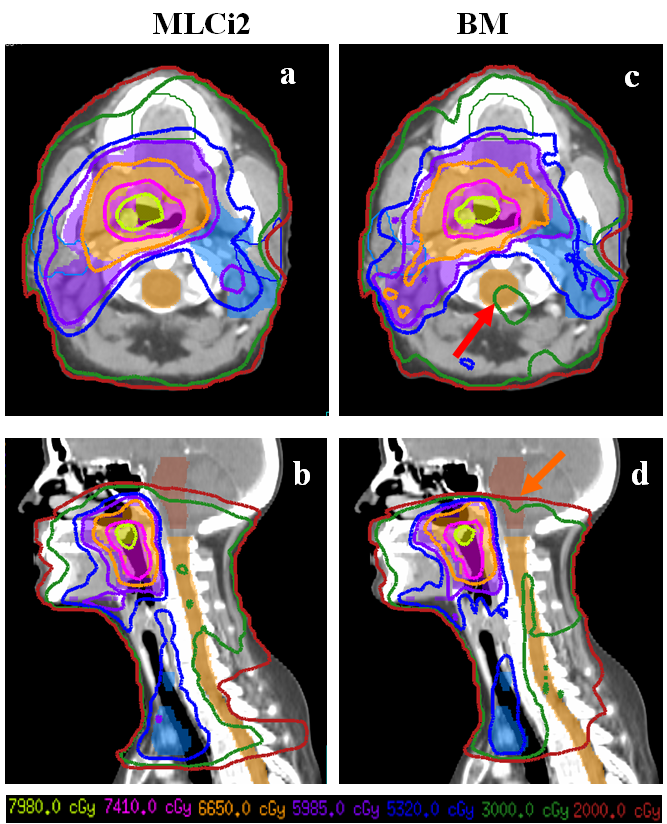


Fig. 1 Dose distributions depending on collimator leaf width: 10 mm MLCI2 leaf width (a, b) and 4 mm BM leaf width (c, d) using a “dose painting” simultaneous integrated boost (DP-SIB) VMAT technique, for a given patient. The PTV56, PTV63, PTV70, PTV78 and PTV84 are defined by the blue, violet, orange, pink and yellow areas respectively. The 95% prescription doses curves corresponding to the different PTVs are the thick lines with the blue, violet, orange, pink and yellow-green colours, respectively. The PTV coverages appear not different. However, BM collimator offers a slightly better dose sparing in the spinal cord (c) (red arrows) and in the brainstem (d) (orange arrows).


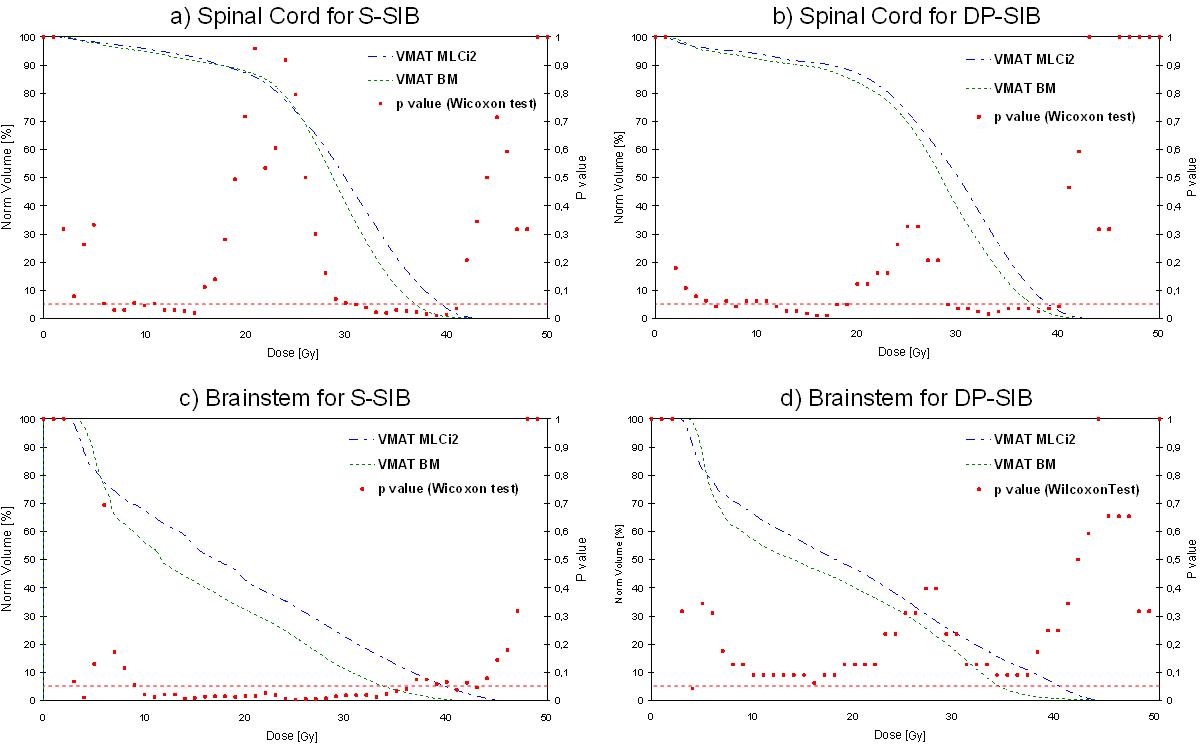


Fig. 2 Spinal cord and brainstem DVHs for collimator leaf width (blue dashed lines 10 mm MLCI2 leaf width; green lines 4 mm BM leaf width) in VMAT technique: Figure a and c with a “standard” simultaneous integrated boost prescription (S-SIB) (mean values for 16 patients) or Figure b and d with a dose painting (DP-SIB) prescription (mean values for 8 patients). Comparison test of the 2 curves has been made by the Wilcoxon test (p values are indicated with red points each Gy).

Table 1. Dosimetric comparison for PTVs between 10 mm MLCI2 leaf width and 4 mm BM leaf width.

|  | **Standard SIB** | | | **Dose painting SIB** | | |
| --- | --- | --- | --- | --- | --- | --- |
|  | MLCi2 | BM | p * | MLCi2 | BM | p * |
|  | **PTV56 (301.3 ± 135.3 cm^3^)** | | | **PTV56 (304.3 ± 124.6 cm^3^)** | | |
| D2% (Gy) | 61.5 ± 2.1 | 60.6 ± 2.2 | 0.01 | 59.7 ± 1.0 | 60.4 ± 1.7 | 0.09 |
| D98% (Gy) | 51.3 ± 1.0 | 51.4 ±0.5 | 0.57 | 51.5 ± 0.6 | 51.2 ± 0.6 | 0.33 |
| Dmean (Gy) | 57.2 ± 0.9 | 56.7 ± 1.0 | 0.01 | 56.3 ± 0.6 | 56.7 ± 0.9 | 0.16 |
| V95% (%) | 95.0 (normalization point) | | / | 95.0 (normalization point) | | / |
|  | **PTV63 (382.3 ± 204.4 cm^3^)** | | | **PTV63 (413.5 ± 180.2 cm^3^)** | | |
| D2% (Gy) | 69.1 ± 1.5 | 68.7 ± 2.0 | 0.11 | 68.7 ± 1.3 | 68.9 ± 1.1 | 0.45 |
| D98% (Gy) | 55.7 ± 3.5 | 55.3 ± 3.4 | 0.80 | 55.6 ± 2.4 | 55.6 ± 1.3 | 0.78 |
| Dmean (Gy) | 63.9 ± 1.3 | 63.6 ± 1.4 | 0.16 | 63.1 ± 1.0 | 63.4 ± 0.8 | 0.33 |
| V95% (%) | 93.6 ± 6.8 | 90.1 ± 5.3 | 0.06 | 88.7 ± 5.7 | 89.7 ± 3.7 | 0.48 |
|  | **PTV70 (170.7 ± 101.8 cm^3^)** | | | **PTV70 (131.4 ± 74.8 cm^3^)** | | |
| D2% (Gy) | 73.5 ± 3.3 | 73.7 ± 2.2 | 0.61 | 75.8 ± 1.6 | 76.2 ± 0.8 | 0.48 |
| D98% (Gy) | 65.9 ± 1.5 | 65.6 ± 1.3 | 0.28 | 65.3 ± 1.4 | 65.8 ± 1.3 | 0.26 |
| Dmean (Gy) | 70.8 ± 1.1 | 70.3 ± 1.5 | 0.10 | 70.5 ± 0.9 | 70.8 ± 0.9 | 0.16 |
| V95% (%) | 96.4 ± 3.2 | 95.3 ± 4.3 | 0.68 | 94.6 ± 3.7 | 96.0 ± 3.2 | 0.33 |
|  |  | | | **PTV78 (18.4 ± 8.8 cm^3^)** | | |
| D2% (Gy) | N/A | N/A | N/A | 80.9 ± 0.9 | 81.2 ± 0.8 | 0.48 |
| D98% (Gy) | N/A | N/A | N/A | 74.3 ± 1.2 | 74.8 ± 1.0 | 0.40 |
| Dmean (Gy) | N/A | N/A | N/A | 77.9 ± 0.8 | 78.1 ± 0.7 | 0.48 |
| V95% (%) | N/A | N/A | N/A | 98.0 ± 1.7 | 98.8 ± 1.1 | 0.16 |
|  |  | | | **PTV84 (20.6 ± 9.4 cm^3^)** | | |
| D2% (Gy) | N/A | N/A | N/A | 85.3 ± 1.1 | 85.6 ± 1.0 | 0.67 |
| D98% (Gy) | N/A | N/A | N/A | 79.3 ± 1.0 | 79.7 ± 0.8 | 0.26 |
| Dmean (Gy) | N/A | N/A | N/A | 82.7 ± 1.0 | 83.1 ± 1.0 | 0.21 |
| V95% (%) | N/A | N/A | N/A | 94.3 ± 4.3 | 96.6 ± 2.7 | 0.12 |

Mean values and standard deviation

The PTVs of lower doses were defined by excluding the PTVs of higher doses.

* p value using wilcoxon test

Table 2. Comparison of dosimetric indexes between 10 mm MLCI2 leaf width and 4 mm BM leaf width.

|  | **Standard SIB** | | | **Dose painting SIB** | | |
| --- | --- | --- | --- | --- | --- | --- |
|  | MLCi2 | BM | p * | MLCi2 | BM | p * |
|  | **56 Gy** | | | **56 Gy** | | |
| HI | 0.127 ± 0.030 | 0.117 ± 0.028 | 0.10 | 0.100 ± 0.014 | 0.109 ± 0.024 | 0.21 |
| CI | 1.363 ± 0.079 | 1.299 ± 0.058 | 0.00 | 1.411 ± 0.103 | 1.327 ± 0.038 | 0.04 |
| COIN | 0.690 ± 0.229 | 0.623 ± 0.258 | 0.28 | 0.339 ± 0.124 | 0.353 ± 0.111 | 0.16 |
|  | **63 Gy** | | | **63 Gy** | | |
| HI | 0.133 ± 0.030 | 0.147 ± 0.031 | 0.01 | 0.148 ± 0.024 | 0.151 ± 0.019 | 0.40 |
| CI | 1.320 ± 0.505 | 1.209 ± 0.302 | 0.03 | 1.130 ± 0.123 | 1.141 ± 0.110 | 0.89 |
| COIN | 0.975 ± 0.568 | 0.880 ± 0.359 | 0.08 | 0.488 ± 0.097 | 0.541 ± 0.107 | 0.36 |
|  | **70 Gy** | | | **70 Gy** | | |
| HI | 0.088 ± 0.020 | 0.093 ± 0.020 | 0.44 | 0.115 ± 0.020 | 0.113 ± 0.009 | 0.48 |
| CI | 1.604 ± 0.520 | 1.442 ± 0.432 | 0.16 | 1.390 ± 0.236 | 1.479 ± 0.276 | 0.16 |
| COIN | 0.577 ± 0.535 | 0.624 ± 0.389 | 0.07 | 0.614 ± 0.114 | 0.565 ± 0.144 | 0.07 |
|  |  |  | **78 Gy** | | | |
| HI | N/A | N/A |  | 0.067 ± 0.008 | 0.068 ± 0.004 | 0.89 |
| CI | N/A | N/A |  | 1.550 ± 0.254 | 1.561 ± 0.255 | 1.00 |
| COIN | N/A | N/A |  | 0.635 ± 0.079 | 0.631 ± 0.079 | 1.00 |
|  |  |  | **84 Gy** | | | |
| HI | N/A | N/A |  | 0.058 ± 0.007 | 0.059 ± 0.007 | 0.67 |
| CI | N/A | N/A |  | 1.177 ± 0.200 | 1.232 ± 0.150 | 0.40 |
| COIN | N/A | N/A |  | 0.766 ± 0.064 | 0.958 ± 0.050 | 0.78 |

Mean values and standard deviation

* p value using wilcoxon test

HI = Homogeneity Index (ideal values = 0)

CI = Conformity Index (ideal values = 1)

COIN = Conformal Index (ideal values = 1)

Table 3. Dosimetric comparison for organ at risk between 10 mm MLCI2 leaf width and 4 mm BM leaf width.

|  | **Standard SIB** | | | **Dose painting SIB** | | |
| --- | --- | --- | --- | --- | --- | --- |
|  | MLCi2 | BM | p* | MLCi2 | BM | p* |
|  | **Spinal Cord** | | | **Spinal Cord** | | |
| D2% (Gy) | 38.3 ± 3.2 | 37.1 ±3.1 | 0.03 | 38.5 ± 2.5 | 37.1 ± 2.5 | 0.16 |
| Dmean (Gy) | 28.7 ± 3.1 | 27.3 ± 2.0 | 0.12 | 28.3 ± 2.5 | 26.7 ± 2.6 | 0.03 |
|  | **Brainstem** | | | **Brainstem** | | |
| D2% (Gy) | 37.2 ± 6.6 | 33.0 ± 7.1 | 0.04 | 34.1 ± 14.2 | 31.3 ± 13.2 | 0.06 |
| Dmean (Gy) | 18.7 ± 6.4 | 15.2 ± 4.7 | 0.00 | 16.8 ± 8.3 | 14.6 ± 7.1 | 0.09 |
|  | **Ipsi-lateral parotid** | | | **Ipsi-lateral parotid** | | |
| Dmean (Gy) | 39.5 ± 10.8 | 40.9 ± 12.0 | 0.47 | 44.0 ± 10.7 | 44.2 ± 10.2 | 0.33 |
| Dmed (Gy) | 39.3 ± 15.7 | 38.7 ± 16.3 | 0.44 | 44.1 ± 13.9 | 45.2 ± 14.2 | 0.16 |
| V15Gy (%) | 80.4 ± 16.7 | 79.0 ± 18.1 | 0.15 | 86.6 ± 15.1 | 86.8 ± 16.2 | 0.60 |
| V30Gy (%) | 62.7 ± 21.2 | 63.5 ± 23.3 | 0.84 | 70.9 ± 22.0 | 70.8 ± 21.6 | 0.58 |
| V45Gy (%) | 46.3 ± 21.2 | 46.9 ± 21.7 | 0.47 | 56.6 ± 23.7 | 56.8 ± 22.3 | 0.89 |
| NTCP** | 0.49 ± 0.24 | 0.48 ± 0.26 | 0.57 | 0.58 ± 0.23 | 0.59 ± 0.23 | 0.33 |
|  | **Contro-lateral parotid** | | | **Contro-lateral parotid** | | |
| Dmean (Gy) | 30.5 ± 2.8 | 30.7 ± 2.6 | 0.57 | 27.8 ± 4.6 | 29.0 ± 2.7 | 0.78 |
| Dmed (Gy) | 27.2 ± 4.9 | 25.7 ± 4.9 | 0.01 | 25.2 ± 5.6 | 23.8 ± 4.3 | 0.16 |
| V15Gy (%) | 66.9 ± 5.4 | 67.3 ± 8.9 | 0.20 | 68.0 ± 11.6 | 69.6 ± 13.9 | 0.16 |
| V30Gy (%) | 46.2 ± 6.2 | 44.6 ± 6.3 | 0.01 | 42.7 ± 9.0 | 41.3 ± 7.5 | 0.21 |
| V45Gy (%) | 29.3 ± 6.8 | 28.5 ± 6.5 | 0.11 | 26.3 ± 8.2 | 26.1 ± 7.3 | 0.67 |
| NTCP** | 0.28 ± 0.06 | 0.27 ± 0.05 | 0.13 | 0.25 ± 0.06 | 0.25 ± 0.05 | 0.67 |
|  | **Healthy Tissue** | | | **Healthy Tissue** | | |
| V5Gy (%) | 79.9 ± 7.9 | 81.5 ± 8.6 | 0.01 | 74.6 ± 10.8 | 76.0 ± 12.0 | 0.12 |
| V10Gy (%) | 64.9 ± 7.9 | 63.4 ± 8.0 | 0.01 | 59.9 ± 8.3 | 58.6 ± 9.4 | 0.26 |
| V25Gy (%) | 33.9 ± 4.5 | 32.9 ± 5.2 | 0.11 | 33.0 ± 4.9 | 31.8 ± 5.5 | 0.26 |
| V50Gy (%) | 5.9 ± 1.5 | 5.0 ± 1.4 | 0.00 | 6.0 ± 1.1 | 5.0 ± 1.1 | 0.04 |
|  | **Skin (3 mm superficial region)** | | | **Skin (3 mm superficial region)** | | |
| Dmean (Gy) | 13.8 ± 2.1 | 13.7 ± 2.4 | 0.12 | 14.0 ± 1.5 | 13.8 ± 1.9 | 0.40 |
| V5Gy (%) | 69.9 ± 6.0 | 69.2 ± 6.0 | 0.16 | 66.5 ± 5.5 | 65.9 ± 6.7 | 0.67 |
| V10Gy (%) | 49.0 ± 6.0 | 48.0 ± 7.2 | 0.15 | 47.8 ± 4.6 | 46.5 ± 6.7 | 0.26 |
| V25Gy (%) | 17.4 ± 53.3 | 16.8 ± 5.7 | 0.06 | 18.9 ± 3.7 | 18.0 ± 4.0 | 0.07 |
| V50Gy (%) | 2.0 ±1.3 | 2.4 ± 1.8 | 0.12 | 2.5 ± 1.2 | 2.9 ± 1.3 | 0.12 |

Mean values and standard deviation

* p value using wilcoxon test

**NTCP: constants for parotid TD50 = 39.9 Gy, n = 1, m = 0.4 defined by Dijkema et al [25; 26].

Table 4. Comparison of efficiency between 10 mm MLCI2 leaf width and 4 mm BM leaf width.

|  | **Standard SIB** | | | **Dose painting SIB** | | |
| --- | --- | --- | --- | --- | --- | --- |
|  | MLCi2 | BM | p * | MLCi2 | BM | p * |
|  | **% of patients with 2 arcs** | | | **% of patients with 2 arcs** | | |
| Percentage (%) | 12.5 | 75 | N/A | 37 | 87.5 | N/A |
|  | **Number of Monitor Units** | | | **Number of Monitor Units** | | |
| Mean values  Standard deviation | 537.8  ± 51.3 | 774.8  ± 151.0 | 0.00 | 548.2  ± 44.9 | 829.5  ± 120.0 | 0.01 |

* p value using Wilcoxon test
